# Feasibility study of the SWITCH implementation process for enhancing school wellness

**DOI:** 10.1186/s12889-018-6024-2

**Published:** 2018-09-14

**Authors:** Senlin Chen, David A. Dzewaltowski, Richard R. Rosenkranz, Lorraine Lanningham-Foster, Spyridoula Vazou, Douglas A. Gentile, Joey A. Lee, Kyle J. Braun, Maren M. Wolff, Gregory J. Welk

**Affiliations:** 1 School of Kinesiology, Louisiana State University, 175C Huey P. Long Field House, Baton Rouge, LA 70803 USA; 2 College of Public Health, University of Nebraska Medical Center, Omaha, NE 68198 USA; 3 Buffett Early Childhood Institute, University of Nebraska, Omaha, NE 68106 USA; 4 Department of Food, Nutrition, Dietetics and Health, Kansas State University, Manhattan, KS 66506 USA; 5 Department of Food Science and Human Nutrition, Iowa State University, Ames, IA 50011 USA; 6 Department of Kinesiology, Iowa State University, Ames, IA 50011 USA; 7 Department of Psychology, Iowa State University, Ames, IA 50011 USA; 8 Department of Health Sciences, University of Colorado at Colorado Springs, Colorado Springs, CO 80918 USA

**Keywords:** Acceptability, Capacity-building, Childhood obesity, Motivational interviewing, Practicality, School engagement

## Abstract

**Background:**

There is a need to identify strategies that enhance the implementation of evidence-based school wellness intervention programs in real-world settings. The present study evaluates the feasibility of empowering school wellness leaders to deliver an evidence-based, childhood obesity-prevention program called *Switch* ™. We specifically evaluated the feasibility of a new implementation framework, based on the robust *Healthy Youth Places* framework, to increase capacity of school leaders to lead school wellness programming.

**Methods:**

The SWITCH (School Wellness Integration Targeting Child Health) implementation process was evaluated in a convenience sample of eight Iowa elementary schools. Teams of three leaders from each school attended an in-person school wellness conference followed by five online webinar sessions delivered by two SWITCH team members. The capacity-building and quality improvement process was designed to empower schools to lead wellness change using methods and concepts from the original 16-week *Switch* ™ program. School wellness leaders completed checklists on two occasions to assess overall school-level implementation as well as setting-level changes in physical education, classrooms, and the lunchroom. Student acceptability of SWITCH was evaluated by the degree of behavior tracking using an online SWITCH Tracker system that promoted self-monitoring. School acceptability and practicality were assessed through an exit survey completed by school leaders.

**Results:**

All school staff reported satisfaction with the SWITCH implementation process. Reports of school- and setting-level implementation were relatively high (2.0 to 2.8 on a 3-point scale) but student engagement, based on use of the online tracking system, varied greatly over time and across schools. Three high implementation schools had average tracking rates exceeding 70% (range: 72–90%) while three low implementation schools had rates lower than 30% (range = 0–23%).

**Conclusions:**

This feasibility study supports the utility of the new implementation framework for promoting school and student engagement with SWITCH. Further testing regarding effectiveness and scale-up of this evidence-based school wellness intervention program is warranted.

## Background

Schools are a frequent target for coordinated health and childhood obesity prevention interventions [1, 2]. However, only a few school-based obesity interventions have shown a significant effect on weight status [2]. Fewer programs have demonstrated sustainability over time, particularly when being generalized to the broader population in less controlled settings [3]. This sustained effectiveness issue has been well characterized in the dissemination and implementation science literature, [4] which has led to calls for research aimed at studying the process of implementing evidence-based interventions within specific settings [5]. The present study fulfills this need by examining the feasibility of empowering school wellness leaders to deliver an evidence-based, childhood obesity-prevention program called *Switch* **™** [6, 7]. The need for feasibility studies is particularly important when a previously evaluated intervention is translated into a new practice system [8]. This is the case with the *Switch* **™** program, which was previously run in schools, with support from YMCA-based community leaders.

The original *Switch* **™** program was developed as a multicomponent, ecologically-based intervention designed to support school wellness programming and contribute to youth obesity prevention [6]. The program capitalized on the coordinated structure and motivation provided through schools, but also targeted the home environment since families exert a direct impact on youth lifestyle behaviors [9]. The integrated program targeted three lifestyle behaviors (physical activity, screen time and fruit/vegetable consumption) in creative ways to help students ‘*switch what they Do, View and Chew*’. The primary behavior change strategies were self-monitoring and goal setting as students were guided to track their lifestyle behaviors over time. The original controlled efficacy study demonstrated significant changes in key behavioral outcome measures and the effects were generally sustained for 6 months following the intervention [7]. Some schools that participated in the original efficacy study continued running the *Switch* **™** program, but the print-based materials made it cost-prohibitive for broader dissemination so subsequent work focused on converting *Switch* **™** to an online platform that could be delivered more efficiently. Formative research by our team demonstrated that a web-based version of *Switch* **™** had similar utility and outcomes compared to the print-based program [10], but simultaneously documented the need to engage and empower school wellness leaders more effectively to take ownership of the programming. Therefore, our priority with a re-branded version of *SWITCH* (*School Wellness Integration Targeting Child Health)* was to promote higher engagement and more effective implementation in schools. Through a project funded by The United States Department of Agriculture (USDA; Grant#: AFRI 2014–08390), we have developed a robust content management system (https://www.iowaswitch.org), resource modules, and an implementation framework to facilitate more effective school wellness programming in schools.

In the present feasibility study, we specifically evaluated the utility of the SWITCH implementation process to increase capacity of school wellness leaders to lead school wellness programming. Based on the robust Healthy Youth Places (HYP) framework [11, 12], the SWITCH implementation process was designed to influence the overall school wellness system, and is consistent with recommendations for *Whole-of-School* approaches to health promotion [13–15]. Schools have strong internal interests in wellness programming, due to documented evidence that it can positively influence academic achievement and other desired school outcomes [14, 16, 17]. Research to date, however, has not adequately explored *how* to operationalize health and wellness programming models to effectively influence the adoption and implementation of these broader school evidence-based programming in schools. Our approach to promoting school wellness change emphasizes the use of equitable community engagement strategies which have been previously supported in the literature [18]. In a systematic review, 16 school-based interventions that targeted diet, physical activity, or anthropometric outcomes were scored on capacity-building and partner involvement [19]. Interventions that built local capacity and created equitable partnerships in schools had greater improvements in health outcomes than interventions that did not embrace these principles to build meaningful partnerships [19].

Consistent with these methods, the SWITCH implementation process was designed to build school capacity and promote autonomy and ownership of school change. Guided by the HYP framework [11, 12], we developed staff development strategies to help schools promote school system change. Resource modules were developed to support programming in different settings (Physical Education [PE], Classroom, and Lunchroom), but the process encouraged adaptation to fit local needs. A customized content management system (www.iowaswitch.org) was also developed to enable school leaders to enroll students, engage colleagues and monitor change in outcomes. However, before initiating broader dissemination of the program and tools, it was important to ensure that the implementation process provided sufficient structure to enable schools to take action. Therefore, the specific purpose of this paper was to evaluate the feasibility of the SWITCH implementation process for building the capacity of school leaders to lead school wellness programming.

## Methods

### Settings and participants

This study took place in eight elementary schools in Iowa, United States, during the spring semester of 2017. The school locations were scattered across the state, and were represented by three town schools, three rural schools, and two suburban schools. The percentage of students eligible for free or reduced price meals ranged from 5.5 to 48.0% (Mean [*M*] ± standard deviation [*SD*] = 23.6 ± 15.6%). These schools mainly enrolled non-Hispanic White/Caucasian students (*M* ± *SD* = 92.1 ± 3.6%, ranging from 86.7 to 96.7%), which is typical for schools in Iowa. Twenty-three (*n* = 23) school staff members participated in the SWITCH implementation process that included capacity-building and quality improvement (i.e., nurses = 7, food service directors = 3, classroom teachers = 6, PE teachers = 3, principals = 2, AmeriCorps/other representative = 2). In addition, 602 students in 4th and 5th grade were asked to complete the SWITCH Tracker (i.e., tracking their daily Do, View, and Chew behaviors online for each week) to capture student engagement. The Iowa State University Institutional Review Board reviewed the study protocol and made an exempt determination for the school-based components of the project since the activities were low minimal and consistent with standard educational practices. Parent consent and student assent were not required since the student data collected through the system were all de-identified and not sensitive in nature.

### The SWITCH implementation framework

The SWITCH implementation framework was based on the established *Healthy Youth Places* [HYP] framework developed by Dzewaltowski and colleagues [11, 12]. The HYP framework follows a community development social ecological systems approach for implementation and has been shown to successfully build the capacity of adult and youth leaders in schools to promote school changes in physical activity [12]. The SWITCH model communicated the school system capacity-building process as a series of *plan-do-study-act* steps following the *Institute of Healthcare Improvement’s* (IHI) group-based rapid cycle improvement model [20].

**Fig. 1:**
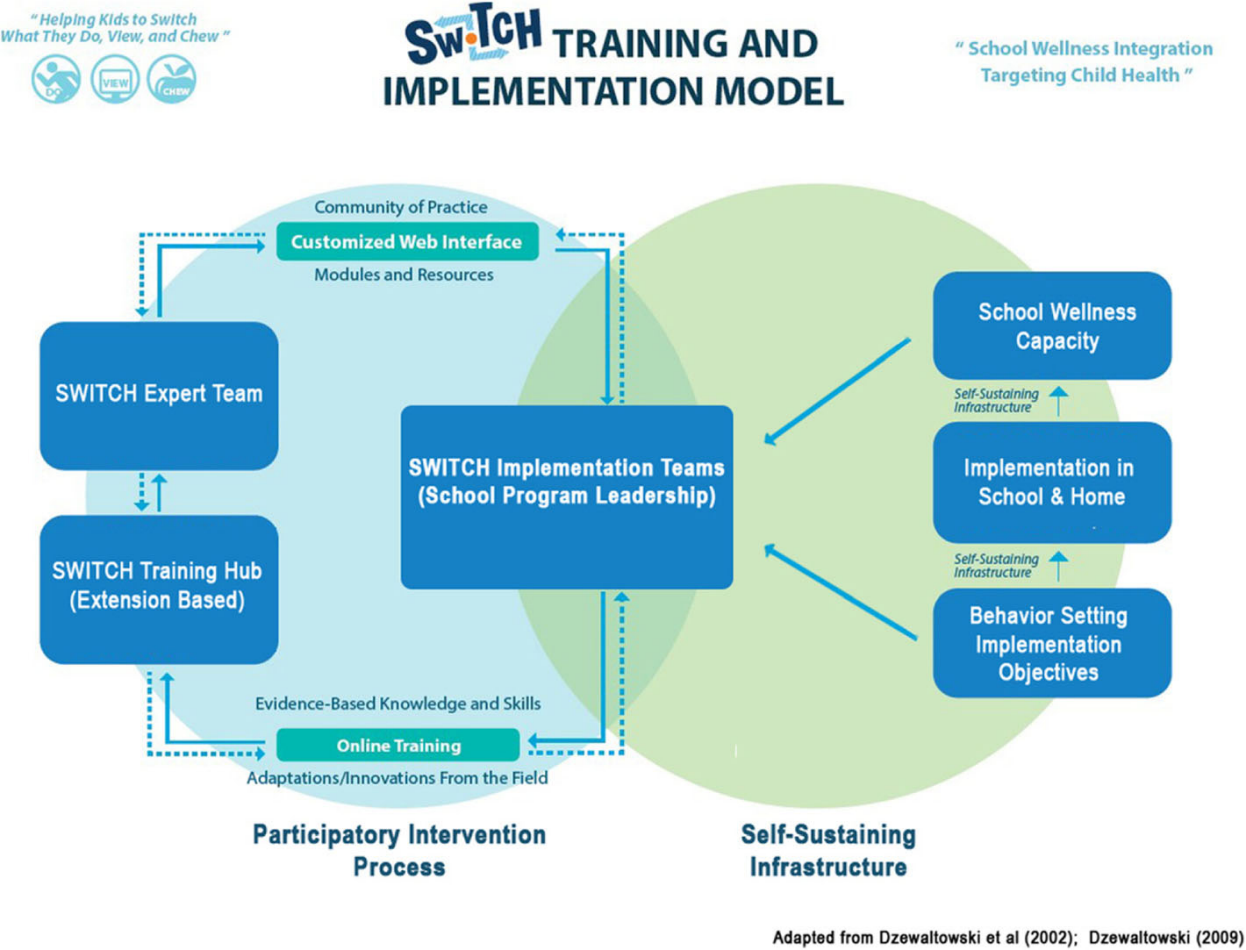
SWITCH Training and Implementation Framework

As shown in Fig. 1, the overall goal of the SWITCH implementation framework was to establish a self-sustaining infrastructure that would empower school teams to implement school wellness practices in creative and customized ways that best fit the current capacity of their school and develop the school’s capacity for enhanced implementation efforts. Therefore, rather than implementing and evaluating a packaged set of intervention materials, the goal was to work with the individual school teams to support integration of *Switch* **™** elements and themes into existing and evolving school wellness practices. The SWITCH capacity-building and quality improvement process included an in-person school wellness conference in the fall 2016 semester, followed by five online webinar sessions, led by two SWITCH team members via GoToWebinar (LogMeIn, Inc., Boston, MA) in the spring 2017 semester. Most webinar conversations were audio-recorded for future coding and analysis.

The initial, in-person SWITCH school wellness conference served as an introduction to the project for participating schools. Each school sent a team of three school leaders to attend the 6-h conference held on the university campus. The schools had freedom to select the 3 leaders from all available staff, but the selected staff typically included nurses, PE teachers, food service personnel, classroom teachers or other staff. Sessions at the conference included updates on USDA local school wellness policy, general strategies for wellness programming, team-building activities and specific information about SWITCH principles and associated resources available to affect different segments of the school environment (i.e., PE, lunchroom, and classroom). A follow-up, 1-h webinar scheduled for a few weeks later addressed questions from the conference and guided schools in how to set up and use the content management system (www.iowaswitch.org) to operationalize SWITCH. Once enabled, this system allowed school wellness leaders to enroll students and to launch the weekly behavior tracking feature mechanism (i.e., SWITCH Trackers) to promote self-monitoring and goal setting for the targeted ‘Do’, ‘View’ and ‘Chew behaviors in SWITCH. This preparatory phase was largely didactic and informational, but it enabled schools to complete internal audits / evaluations and to prepare for planning and goal setting.

A set of *quality elements* were provided to summarize recommended implementation strategies for the three leaders guiding change in their school: 1. Meet weekly about wellness strategies and planning, 2. Facilitate online tracking of student behaviors, 3. Facilitate the distribution of incentives for student engagement, 4. Communicate wellness efforts to parents, and 5. Use promotional materials within school setting to promote awareness. A set of *best practices* were also provided to outline recommended strategies to promote engagement in specific school settings (PE, lunchroom, and classrooms): 1. Use the SWITCH modules or related activities, 2. Use the interactive SWITCH posters to reinforce lessons, and 3. Engage in the SWITCH Community of Practice website (https://switch.ning.com) to share information across and between schools. Although schools were provided with training and resources, they had complete autonomy in how the quality elements and best practices were implemented in their schools.

After schools initiated programming in February, a series of capacity-building and quality improvement webinars (approximately monthly) were used to promote and check in on implementation over the 12-week implementation phase. A novel aspect of the SWITCH implementation framework is that it utilizes principles of motivational interviewing (MI) to promote autonomy and goal setting [21, 22]. An advantage of MI for behavior change is that it helps individuals overcome ambivalence to change and it may provide a similar advantage at the organizational level for prompting action in a supportive and encouraging way. While the utility of MI for promoting individual behavior change is well documented [21, 23], it has not previously been used for promoting equitable collaboration and *systems* change in school settings. Therefore, an important component of our feasibility evaluation was to examine the utility of this approach for promoting engagement and building capacity in schools. Two SWITCH team facilitators, one of whom had extensive training and practice of MI, used MI-enhanced conversations to help schools evaluate their progress and set future goals and action plans. During each webinar session, schools were asked to report about which implementation efforts (quality elements and best practices) they believed were going well and why, and which were not going so well and why. The SWITCH team facilitators guided discussion for overcoming implementation barriers based on the difficulties reported by the schools and provided resources or ideas when appropriate. Following the MI-enhanced conversation, the SWITCH team facilitators invited the school leaders to set 1–2 goals for the next month. These goals could target specific SWITCH quality elements and best practices, or any aspect of school wellness that the school leaders were interested in prioritizing. In subsequent calls, discussion of goal progress and implementation led off the webinar sessions followed by the invitation for schools to set new goals to pursue over the next month. This process was used to help schools employ the ‘plan-do-study-act’ cycle and to embrace use of continuous quality improvement strategies.

At the end of the 12-week implementation phase, a final wrap-up webinar was conducted to capture the school teams’ overall perceptions about SWITCH and their experiences with the process. In addition, a satisfaction survey was also used at the end of the implementation phase to capture feedback from school wellness leaders to understand schools’ satisfaction with SWITCH and to provide additional insight about strategies for improving SWITCH in the future.

### Measures and evaluation plan

#### Evaluation of equitable collaboration

A process evaluation of the SWITCH implementation framework was conducted to evaluate the utility of following an equitable collaboration process using MI-based strategies. The Motivational Interviewing Treatment Integrity (MITI) scale was used to specifically assess the adherence to MI principles during the middle three webinar sessions. An MI-trained analyst provided subjective ratings capturing the degree of adherence to MI principles, with each scored on a five-point Likert scale (1 = minimum; 5 = maximum). Four global scores were calculated to determine the extensiveness of MI elements in the webinar sessions (c*ultivating change talk*, s*oftening sustain talk, partnership,* and e*mpathy).* In addition, two MI summary scores were calculated: *technical skills* (the average of c*ultivating change talk* and s*oftening sustain talk*) and *relational skills* (the average of *partnership* and *empathy*). The MITI has been previously validated and has been used in clinical and public health research [21, 23].

#### Indicators of school, setting and student implementation

The utility of the SWITCH implementation framework was evaluated indirectly by assessing whether schools could independently run SWITCH on their own. Indicators of school and setting implementation were also obtained from two checkpoint surveys completed by the school wellness leaders during the 12-week implementation phase (at approximately weeks 4 and 8). These surveys were used in a formative way to evaluate the degree to which schools followed the SWITCH ‘quality elements’ for overall school implementation as well as adoption of ‘best practices’ in the three targeted settings (classrooms, PE, and lunchroom). School teams self-reported the degree to which they followed the five quality elements using a 3-point scale (rarely/never = 1, sometimes = 2, and often/always = 3). The mean score on the five items was used to represent school implementation. School wellness leaders also self-reported the degree to which they followed best practices within each of the targeted settings: classroom (2 items, with ratings made for each classroom teacher), PE (5 items) and lunchroom (5 items). Mean scores were computed for each of the three settings and for the capacity of school wellness team, which were subsequently averaged to create an indicator of school-level program implementation.

Student implementation was assessed by recording student utilization of the web-based SWITCH tracking system (i.e., the SWITCH Trackers). Student self-monitoring of Do, View, and Chew behaviors is a key mediating variable in the program, so emphasis in the process was placed on helping schools to promote utilization of the online tracking system by the students. School wellness leaders used a variety of strategies to promote, encourage, and/or facilitate tracking so it is a useful indicator of both student acceptability and implementation. The 12-week SWITCH program rotated through four (3-week) cycles of Do, View and Chew themes, so students had opportunities to track each of the targeted behaviors four times throughout SWITCH. The percentage of completed Trackers was computed for each student, and then aggregated at the (class and) school level. Descriptive analyses examined the overall use of Trackers over time, as well as variability in Tracker completion rates by school.

#### School satisfaction with SWITCH

School satisfaction with the SWITCH implementation process and programming were captured using an exit survey that included a series of 18 questions in four sections: school implementation of SWITCH (4 questions), SWITCH support and engagement (3 questions), satisfaction in SWITCH program and delivery (5 questions), and feedback about online components of the SWITCH program (6 questions). The satisfaction-related questions asked the school wellness leaders about their levels of satisfaction with the capacity-building and quality improvement sessions, their perceptions of the overall implementation process at their school, as well as suggestions for future improvement. The primary questions had three choices (i.e., 1 = *not satisfied,* 2 = *somewhat satisfied*, 3 = *very satisfied)* but respondents could provide additional comments and suggestions through open-ended text boxes. The exit satisfaction survey was distributed to the school wellness leaders at the end of the project through Qualtrics.com. Descriptive analyses were conducted to summarize school satisfaction with SWITCH programming. The survey was also used to facilitate conversations and open-ended feedback during a closing interview. The qualitative notes from this session were used to summarize the engagement of the school wellness leaders and provide additional feedback about the program delivery process.

## Results

The study was designed to evaluate the feasibility of the SWITCH implementation framework for promoting implementation of school wellness programming in schools. An evaluation of the SWITCH implementation framework is provided first, followed by indicators of school-, setting- and student-level implementation. Results from a satisfaction survey provide insights about the school’s satisfaction with the overall process.

### Evaluation of MI strategies for promoting equitable collaboration

The utility of our implementation framework was more directly evaluated with data obtained during the webinar sessions which were designed to build capacity in school teams. All schools participated in the webinar sessions, except for one case at the final webinar session where one school did not participate. Table 1 shows the MITI-related results based on the recordings. MI global scores for sessions employing MI (i.e., sessions 2, 3, and 4) ranged from 4.0 to 5.0 while scores for the more didactic session (i.e., session 1) ranged from 1.0 to 2.0. Similarly, MI summary scores (i.e., Technical Global and Relational Global) for the middle three sessions (ranging from 4.3 to 5.0) also showed considerably larger means than the first session. The MI-enhanced sessions (i.e., sessions 2, 3, and 4) were found to include the intended elements of MI to a greater extent than the session that did not use this method (i.e., session 1). Session 5 was not analyzed as no MI-enhanced conversations took place.

**Table 1:** Descriptive Results for the MI Variables across the Webinar Sessions

MI Variables	Session 1 (*n* = 3)	Session 2 (*n* = 5)	Session 3 (*n* = 5)	Session 4 (*n* = 4)
Global Scores of MI Principles				
Cultivating Change Talk	1.0 ± 0	4.4 ± .5	4.6 ± .5	5.0 ± 0
Softening Sustain Talk	2.0 ± 0	4.2 ± .4	4.0 ± .7	5.0 ± 0
Partnership	2.0 ± 0	5.0 ± 0	4.8 ± .4	4.8 ± .5
Empathy	1.7 ± .6	4.2 ± .4	4.4 ± .5	4.3 ± .5
MI Summary Scores				
Technical Global	1.5 ± 0	4.3 ± .3	4.3 ± .6	5.0 ± 0
Relational Global	1.8 ± .3	4.6 ± .3	4.6 ± .4	4.5 ± 0

### School, setting and student wellness implementation

The utility of the SWITCH implementation framework is indirectly evidenced by the ability of school teams to initiate and run SWITCH on their own. Following the two online training and capacity-building sessions, schools were able to use the customized SWITCH content management system to add classes and enroll students into the program. Schools all successfully guided students through the behavioral assessments and initiated student use of the behavioral tracking system to prompt awareness and behavior change.

The school and setting implementation evaluation captured the degree with which schools adhered to the SWITCH quality elements and the degree with which they followed the SWITCH best practices for wellness programming in the classroom, PE, and lunchroom settings. Table 2 below shows good adherence to the quality elements, with overall school implementation mean scores ranging from 2.0 to 2.8 on a three-point scale. The reported compliance with the setting-specific best practices had similar distributions (Classroom: 2.6 ± 0.3; PE: 2.3 ± 0.5; Lunchroom: 2.6 ± 0.3). Schools 2, 3, and 5 demonstrated higher levels of implementation than other schools, while schools 4 and 7 showed lowest levels of implementation.

**Table 2:** School and Setting Level Implementations of the SWITCH Programming (*n* = 23)

Implementation Venues	1	2	3	4	5	6	7	8	Overall
School Implementation	2.6	2.8	2.8	2.0	2.8	2.6	2.1	2.7	2.6 ± 0.3
Classroom	2.8	2.9	2.4	2.3	2.8	2.2	2.4	3.0	2.6 ± 0.3
Physical Education	1.6	2.7	2.5	2.7	2.5	2.0	1.6	2.8	2.3 ± 0.5
Lunchroom	2.6	2.7	3.0	2.2	2.7	2.2	2.5	2.8	2.6 ± 0.3

**Fig. 2:**
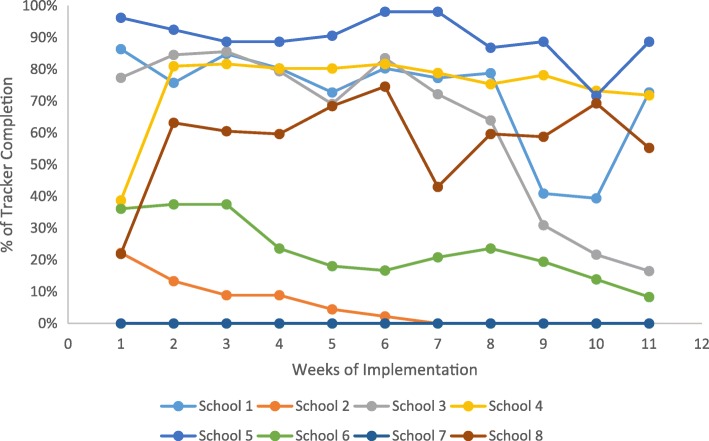
Students’ Weekly SWITCH Tracker Completion Rate across the Schools

The indicator of student acceptability and implementation was the aggregated completion rates of students tracking their Do, View, and Chew behaviors through the SWITCH content management system (www.iowaswitch.org). The evaluation revealed considerable variability in the Tracker completion rates across the schools with some schools having extremely high rates (viz., schools 1, 3, 4, 5) and some schools showing lower completion rates (viz., schools 2, 6) or not engaging in it at all (viz., school 7; See Fig. 2). Figure 2 also depicts the distinct trend of behavior tracking between the schools. Some schools maintained relatively high completion rates over time (e.g., schools 4 and 5), while other schools showed variable patterns of completion (viz., schools 3 and 8). Two other schools exhibited declines in engagement over time (schools 2 and 6). The between-school variation of Tracker completion rates generally coincided with the pattern of the school- and setting-level implementation results described above. Schools with lower levels of SWITCH program implementation were less likely to engage students in behavior tracking activities, hence the lower Tracker completion rates. In addition, qualitative observations based on the webinar sessions provided insight into the variability. Rates of implementation were highest in schools that incorporated specific time in the school day for youth to complete Trackers at school. This variability provides some support for the improved efficacy for the redesigned program and the motivational interviewing procedures, as the original *Switch* ™ implementation generally saw lessening engagement across all schools over the duration of the program.

### School satisfaction

Satisfaction surveys revealed at least moderate satisfaction with the SWITCH implementation process prior to launch (*M* ± *SD* = 2.5 ± 0.5; on a 5-point scale) and with the monthly webinar sessions during the implementation (*M* ± *SD* = 2.4 ± 0.5). The school implementation teams reported the webinar sessions promoted team cohesion in implementing the program (*M* ± *SD* = 2.6 ± 0.6). The school SWITCH teams also provided constructive feedback and advice on how to improve the materials and how to enhance the capacity-building and quality improvement sessions.

The school wellness leaders had favorable comments about the SWITCH modules (classroom, PE, and lunchroom) but they also reported that the materials could be more user-friendly and less time consuming, and that some activities and lessons needed to be further revised to better fit students’ developmental levels. For example, in the SWTCH Classroom module, although teachers had autonomy in selecting which classroom activities/lessons to complete, some teachers reported that they would have preferred to have a list directing them to specific activities/lessons. Regarding the SWITCH PE module, some PE teachers reported that implementing the “full” SWITCH PE Module would be difficult, because it would require replacing their already planned curriculum for a semester. In these cases, PE teachers often utilized the SWITCH PE “warm-up” module, which was reported as being easier to implement because it did not require an overhaul of already planned curriculum, while helping to integrate components of health education related to the SWITCH behaviors into the PE setting. For the SWITCH Lunchroom module, schools that did not have representation from this setting reported that implementation and buy-in was difficult to achieve. This highlights the importance of the recommendation to include representation from each school setting on schools’ SWITCH implementation teams.

Generally, the schools preferred shorter online training webinar sessions (i.e., less than 1-h), online resources (such as content that could be viewed independently), and webinar times that better fit the school teams’ schedules (i.e. offered directly before or after school on preferred days). Although the sessions were reported as being highly useful for helping schools to be accountable for making progress, for helping to determine needs, and for developing action steps and goals, staff time was reported as being very limited. A major complicating factor was the challenge of finding times that worked for three school staff members to attend monthly webinars. Similarly, the school wellness leaders also reported that meeting regularly as a team (approximately weekly) was also difficult to implement in practice due to conflicting schedules and limited “free time”. In addition, school teams reported not having a clear outline for how to go about leading and planning meetings from week to week. Future iterations of SWITCH will better prepare school teams with information and agendas to facilitate SWITCH implementation.

The facilitated interviews that took place after the conclusion of SWITCH revealed possible barriers to implementation of SWITCH in schools. A lack of administrative ‘buy-in’ for SWITCH or setting-level support (classroom, PE, or lunchroom) for implementing quality elements and best practices were reported as barriers to effective implementation. Although administrators were required to provide their signature to enroll in SWITCH, it may be that competing demands or interest (e.g., standardized testing, curriculum needs, etc.) may have re-directed administrator’s commitment to initiating and implementing SWITCH. It could also be that administrators did not fully comprehend the comprehensive nature of SWITCH and the degree of commitment that full implementation would require. Schools are institutionalized settings with pressures for competing curricula or subjects and mandatory standardized assessments. Staffing and time were reported as two major constraints for schools to fully adopt and implement an outside programming.

## Discussion

The present study was designed to evaluate the feasibility of the SWITCH implementation framework that utilizes a *Whole-of-School* approach, equitable collaboration, and a capacity-building and quality improvement process for promoting effective implementation of the evidence-based SWITCH wellness programming in schools. Consistent with recommendations [8], we examined the utility of this new distributed online implementation framework, as well as SWITCH implementation in schools to evaluate feasibility. Although there are numerous examples of school-based health and wellness intervention approaches [24–27], many studies employ highly standardized intervention protocols and delivery methods without allowing or promoting local adaptation of the intervention components [19, 28]. Our approach is similar to the WHO Health Promoting Schools framework [28] in that it aims to address the implicit health-related values and attitudes present within the school setting, as well as to engage with family and community influences on children’s health and well-being. One key area where our approach differs is that SWITCH does not rely on a standardized health education curriculum.

There are few examples of related studies but one is an implementation evaluation of the Fuel Up to Play 60 (FUTP60) program by Hoelscher et al. [24]. Like the original *Switch™* program, FUTP60 provides school wellness leaders with a range of activities to promote opportunities for physical activity and healthy eating through school. Schools often lack the capacity and infrastructure, however, to carry out programming without some facilitation and support. In the FUTP60 evaluation, representatives from the Dairy Council provided logistical support and guided a program advisor within the school to promote implementation. The SWITCH implementation framework is similar to FUTP60, but relies on teams of three school wellness leaders to work together to promote school system change. In contrast to the use of social marketing elements that leverage professional football player role models to motivate and engage youth, the SWITCH approach instead focuses on a process to boost motivation and capacity-building among school wellness leaders to foster health and wellbeing in youth.

Consistent with HYP and IHI implementation frameworks, our revised SWITCH implementation framework was conceptualized as a standardized capacity-building and quality improvement process, rather than a standardized curriculum or set of materials [11, 12, 29]. Through the webinar sessions, schools were provided with guidelines for effective implementation (‘quality elements’), but they were given flexibility in how to carry out the programming in their own preferred way. Implementation was facilitated with the use of a content management system that allowed students to complete online tracking of their Do, View, and Chew behaviors (www.iowaswitch.org). School implementation teams were provided with curriculum resources and posters to complement programming, but they had latitude to decide how these program materials would be used. The standardized capacity-building process ensured that the approach could be systematically evaluated, while the flexible implementation enabled the intervention to be tailored and customized to fit local needs and interests. According to the tenets of self-determination theory [30], promoting competence and providing school stakeholders with autonomy over the local implementation process are important in building sustainable capacity and motivation for on-going wellness promotion efforts within the school setting.

In this study, there was diversity of roles among the school stakeholders who took part in the coordinated efforts to build capacity of schools to plan, implement and sustain evidence-based school wellness practices. The most highly represented role was school nurses, who frequently take on health-related responsibilities, and are often seen as role models for health promotion in the school setting [31]. Aside from nurses, classroom teachers, food service directors, principals, and PE teachers were also represented, although fewer took on leadership roles within schools’ core teams. The USDA Final Rule on School Wellness Policies requires wellness policy leadership of at least one school official who has authority and responsibility to ensure that each school complies with wellness policy [32], but the rule is not prescriptive regarding what roles are best suited for these leadership positions.

During the SWITCH webinar sessions, the school wellness leaders are guided through self-assessments of their school wellness environments and student health behaviors to provide a comprehensive school wellness report that is individualized to their school. This feedback provides school wellness leaders and school administrators a summary of their investment in school wellness and whether or not it has resulted in positive changes, or if alternative modifications are needed. Undergoing such a process provides valuable information that could be used to guide wellness promotion efforts, identify strengths, and evaluate whether changes were effective over time.

Based on the implementation data, our evaluation supports the feasibility of implementing SWITCH through this distributed implementation framework. The overall indicators of school-level implementation was moderate to high (ranging from 2.0 to 2.8 on a 3-point scale) but variations were obvious across the schools. The high engagement by some schools is quite encouraging, since schools were tasked with carrying out SWITCH completely on their own, and most were highly successful. The variability shows that some schools were more effective at implementation than others, and future work is needed to understand the factors that influence the variability. Such heterogeneity of response may be useful from an evaluation perspective, since it allows us to examine whether implementation influences outcomes. The higher school-level implementation was reflected in higher implementations in the three specific settings (i.e., PE, classroom, and lunchroom). These setting-level implementation data (see Table 2) provide useful feedback for both the research team as well as the school implementation teams to identify barriers and issues that should be addressed in future SWITCH initiative evaluations. Because schools were asked to self-report their levels of implementation, however, more objective process data (e.g., through random field observations) would be needed to accurately capture both school- and setting-level implementations in future evaluations. For example, within a school, the wellness leaders may have felt that they were following the recommendations, but there was likely considerably more variability across the schools in the way that the project was carried out. This was evident in the highly variable completion rates of the web-based behavior tracking by the students. While this indicator was used to reflect student engagement, it is also a reasonable indicator of overall school implementation, since schools had to work within their system to help ensure that students had opportunities to access computers and interact with the website to track. Promoting completion of the weekly Trackers is important in empowering change in the students, since it provides a way for students to learn about their lifestyle behavior through the context of SWITCH. The impact of this variability in school, class and individual tracking will be further examined in future studies.

The use of motivational interviewing (MI) proved to have good utility for capacity-building and promoting equitable collaboration, autonomy, and ownership within the schools. To our knowledge, the use of MI for creating organizational change has not been previously reported. Most applications of MI are in individual counseling settings, where the focus is on promoting health behavior change [21, 23], but this project demonstrates the potential of MI for promoting school system change through group-based intervention sessions. Specifically, our results demonstrated that MI elements were successfully infused into the targeted webinar sessions to empower schools to implement the SWITCH initiative in their schools. Although the school and student engagement varied over time and across schools or settings, the MI-enhanced session model displayed good utility in eliciting organizational changes in schools for enhanced wellness. It was not possible to directly evaluate the impact of the MI-enhanced sessions in the project since all schools received the same support. However, overall feedback on the sessions was very positive and it was evident from the discussions that schools valued the autonomy to run the project on their own. The formalized use of MI will be further investigated in future evaluations of the SWITCH initiative.

Our school surveys showed general satisfaction with implementation activities and with the SWITCH components, but refinements will be needed to facilitate seamless and widespread adoption of the initiative. From the data we collected, it appears that better implementation methods and additional refinements to enhance the website are needed. Obtaining additional and on-going school stakeholder input will be necessary to maximize the utility and effectiveness of the SWITCH initiative.

## Limitations

Like all research, this project has some limitations. Given the first priority to investigate feasibility, we started with a relatively small sample size of schools, although our ambition is to scale up in coming years in an effort to have a population effect. The schools currently taking part in SWITCH were early adopters, and might have school wellness leaders who were more motivated to address wellness than would be typical of most Iowa schools. Another limitation lies in the fact that we were only able to analyze randomly selected segments of the webinar sessions (~ 20 min per session). Analyzing these audio-recorded conversations was time-consuming. Therefore, the MITI results may not truly reflect the entire presence of MI principles in full conversations. Another limitation was having only one person analyze the MI data. Using a second analyst and computing the inter-rater reliability would have allowed us to determine the data reliability. However, the MI analyst received intensive training sessions and was knowledgeable on the coding task. An additional limitation of the study lies in the use of self-report measures for the wellness leaders to rate SWITCH implementation in their schools. To minimize social desirability bias [33], it would be useful to adopt a more objective measure to capture school and setting implementation (e.g., field observations) in future evaluations. The other limitation of the study lies in the fact that students’ behavior tracking as an important factor of SWITCH was not reinforced in all schools. In particular, one school completely skipped this element in their implementation efforts, and two additional schools showed low levels of Tracker completion rates. Although the intention of this present SWITCH implementation framework investigation was to empower schools to implement the initiative in their own preferred way, the research team could have encouraged the schools to adopt the online behavioral tracking among their students.

## Conclusion

The School Wellness Integration Targeting Child Health (SWITCH) implementation framework is uniquely designed to build the capacity of schools to plan, implement, and sustain evidence-based school wellness practices, while simultaneously providing resources and opportunities for schools to meet requirements set forth by the USDA’s Final Rule on School Wellness Policies [32]. The original *Switch*™ was an evidence-based program, from which the updated SWITCH model has evolved. Although we still refer to SWITCH as a program, SWITCH has been conceptualized as a standardized implementation process, rather than a curricular program, where school capacity is built through quality improvement cycles, staff development, and provision of helpful resources. The SWITCH implementation process promotes a standard set of best practices and quality elements that are not standardized in their application across schools, but are adapted to local conditions by stakeholders within each school. The over-arching goal of SWITCH is to develop a *Whole-of-School* infrastructure that is self-sustaining, and enables school wellness leaders to implement evidence-based wellness practices that are both culturally consistent and that meet local needs. The framework makes innovative use of a motivational interviewing process for collaborating with school wellness leaders to promote self-determined motivation, addressing important motivational factors such as autonomy, competence, and relatedness [30]. Such theory-based features of the framework should allow for greater value investment on the part of school wellness leaders, potentially leading to better long-term sustainability of wellness promotion efforts in the schools. Thus far, the feasibility of the SWITCH implementation framework shows promise, as the components and activities were accessible and acceptable to our sample of the target population. Although school wellness leaders reported adequate satisfaction with SWITCH, further refinements will be needed to improve the efficiency and to maximize engagement with SWITCH intervention efforts. These future refinements should allow for and accommodate local characteristics of schools with great variations of students and wellness leaders. Our results to date suggest that motivational interviewing sessions can be used to empower schools to adopt and implement the SWITCH initiative in their schools as we expand SWITCH at scale in the coming years. Further testing of effectiveness and scale-up of this evidence-based school wellness promotion intervention are warranted.

## Abbreviations

FUTP60: Fuel Up to Play 60
HYP: Healthy Youth Places
IHI: Institute for Healthcare Improvement
MI: Motivational Interviewing
MITI: Motivational Interviewing Treatment Integrity
PE: Physical Education
SWITCH: School Wellness Integration Targeting Child Health
USDA: United States Department of Agriculture
WHO: World Health Organization
YMCA: Young Men’s Christian Association
